# A General Model for Binary Cell Fate Decision Gene Circuits with Degeneracy: Indeterminacy and Switch Behavior in the Absence of Cooperativity

**DOI:** 10.1371/journal.pone.0019358

**Published:** 2011-05-19

**Authors:** Mircea Andrecut, Julianne D. Halley, David A. Winkler, Sui Huang

**Affiliations:** 1 Institute for Biocomplexity and Informatics, University of Calgary, Calgary, Alberta, Canada; 2 Commonwealth Scientific and Industrial Research Organisation (CSIRO) Materials Science and Engineering, Clayton, Australia; 3 Monash Institute for Pharmaceutical Science, Parkville, Australia; University of Nottingham, United Kingdom

## Abstract

**Background:**

The gene regulatory circuit motif in which two opposing fate-determining transcription factors inhibit each other but activate themselves has been used in mathematical models of binary cell fate decisions in multipotent stem or progenitor cells. This simple circuit can generate multistability and explains the symmetric “poised” precursor state in which both factors are present in the cell at equal amounts as well as the resolution of this indeterminate state as the cell commits to either cell fate characterized by an asymmetric expression pattern of the two factors. This establishes the two alternative stable attractors that represent the two fate options. It has been debated whether cooperativity of molecular interactions is necessary to produce such multistability.

**Principal Findings:**

Here we take a general modeling approach and argue that this question is not relevant. We show that non-linearity can arise in two distinct models in which no explicit interaction between the two factors is assumed and that distinct chemical reaction kinetic formalisms can lead to the same (generic) dynamical system form. Moreover, we describe a novel type of bifurcation that produces a degenerate steady state that can explain the metastable state of indeterminacy prior to cell fate decision-making and is consistent with biological observations.

**Conclusion:**

The general model presented here thus offers a novel principle for linking regulatory circuits with the state of indeterminacy characteristic of multipotent (stem) cells.

## Introduction

Development of the diversity of cell types in the mammalian body involves pluri- and multipotent stem and progenitor cells making fate decisions that are typically binary in nature, thereby committing to either of two to distinct cell lineages [1], [2], [3]. The appropriate lineage-specific genes that implement the phenotypes of the various cell types have to be either induced or suppressed in order to ultimately produce the genome-wide gene expression patterns commensurate to a particular cell lineage [4], [5]. Cell fate (lineage)-determining transcription factors (TFs) that directly control the expression of these lineage-specific genes also play a central role in coordinating entire gene expression programs, for instance, ensuring their mutual exclusivity, by engaging in specific gene regulatory circuits [6], [7], [8], [9]. Over the past years an increasing number of such gene circuits that govern the binary decisions in which a pluri- or multi-potent cell faces the choice of committing to two mutually exclusive lineages have been characterized, ranging from circuits of embryonic stem cells to those in various adult multipotent progenitor cells that control the choice between two alternative differentiation options [2], [3], [10], [11].

From the cases studied the picture is emerging that the typical architecture of the core gene regulatory circuit that drives the binary lineage splitting in a common precursor consists of at least of two mutually repressing (cross-antagonizing) TFs, *X* and *Y*, each of which is typically a fate determining TF for either one of the two mutually exclusive lineages and is later expressed as a lineage-specific marker at high levels. Fate determining TFs are sufficient to impose a lineage decision and much of the ensuing canonical cell phenotype if over expressed in the precursor cell (or even in cells of related lineages, leading to ‘reprogramming’) [12], [13].

Dynamically, the mutual repression of the two TFs *X*, and *Y* has long been proposed to establish a bistable “toggle switch”, readily explaining the two mutual exclusive fate outcomes characterized by the stable expression configuration {*X*>>*Y*} and {*Y*>>*X*}, respectively [2], [14], [15], [16], [17]. Conversely, the multipotent progenitor or stem cell is in a metastable state of indeterminacy, poised to commit to either lineage depending on instructive signals or stochastic influences that will elevate the expression of either *X* or *Y*. They express the “promiscuous” expression pattern {*X*≈*Y*} in which both opposing TF are present at low levels and which is characteristic of multipotent cells. This undetermined state is locally stable, consistent with the notion of a “ground state” of the ES cells [18] but is globally rather unstable because many perturbations (such as just a suboptimal culture conditions) enforce a fate decision and commitment into one of the two available fate options: the {*X*≈*Y*} balance is tilted into the stable {*X*>>*Y*} or {*Y*>>*X*} state, and hence, it is often considered “metastable” [9], [19]. The differentiated states are more stable, as reflected in the quasi-irreversibility of terminal differentiation. Of interest, the extent of stability of the poised metastable {*X*≈*Y*} state appears to be regulated by signaling pathways [18], [20].

Thus, this simple model system has (at least) three experimentally observable attractor states, two stable asymmetrical attractor states (with steady-state patterns {*X*>>*Y*} and {*Y*>>*X*} and a central metastable {*X*≈*Y*}. It has been suggested that the latter (meta-) stable state, which represents the poised stem cell state, is stabilized by the auto-stimulation of the TFs *X* and *Y*. In fact for numerous circuits that control binary cell fate decisions, there is functional and molecular evidence for a circuit architecture that would support cross-antagonism combined with self-activation [3], [7], [9], [10], [21]. Examples include the GATA1-PU.1 circuit [3] in the common myeloid progenitor (CMP) cell which faces the fate options of the erythroid and the myeloid lineage, the PU.1-c-Jun and the PU.1-C/EBP αcircuits [22], [23], [24], [25], [26] which control the decision between the neutrophil and the monocyte/macrophage cells, or in the early embryo, the Oct4-Cdx2 and the Nanog-Cdx2 circuit which face the decision between the pluripotent inner cell mass and the trophoectoderm cells [27], [28]. Typically, the functional evidence arises from the observation that overexpression of one of the TFs, e.g., *X*, results in the down regulation or reduced activity of *Y*. Support for the role of self-activation is weaker; evidence is often provided indirectly by the presence of binding sites for *X* in the promoter of *X* and in some case by promoter reporter studies.

The central problem for mathematical modeling is to formulate a dynamical model based on what is known about molecular interactions and to show that it predicts the three attractor states. Models are typically formalized as chemical rate equation in ideal chemical reaction conditions. The chemical kinetics formalism corresponds to “stocks and flows” models of systems theory [29] and is based on the laws of mass action in chemistry. One source of potential confusion is that herein ‘connections’ of the circuit (edges of reaction networks) represent a physical network of reactant transformation ( = flows), that are subjected to conservation of matter at each network node ( = stock) but that this network is then mapped to an abstract dynamical system which fundamentally represents a different class of networks, namely a causal *influence network* free of mass preservation constraints and flows. Thus, these more abstract dynamical system networks constitute a coarse-grained model of the chemical reactions. This mapping between a physical system of chemical reactions and a formal, minimal dynamical system is often taken for granted. Often these two levels of description are not even distinguished. Importantly, there is no 1∶1 mapping between these two descriptions, which is important to keep in mind when detailed information about the vastly complicated underlying chemistry is lacking. More specifically, in our circuit treated as a dynamical system the key question is how the two inputs of each circuit node, *X* and *Y* are integrated to influence the output (rate of change of the value of *X* or *Y*) for which information about molecular events that is needed for a formulating a precise chemical equation is absent.

The problem of mapping between these two levels of description is often manifest in the interpretation of the steep sigmoidal “transfer function” (characterizing how the input variable *X* regulate the rate of change (d*Y*/*dt*) of its molecular target. Dynamical systems considerations require such sigmoidality for producing multi-stability in deterministic systems. Sigmoidal functions are often by default equated with “cooperativity”. Herein lies a potential for misunderstanding. Thus, let as refer a sigmoidal transfer function in system equations as “*functional* cooperativity” to distinguish it from the actual “*molecular* cooperativity” which was historically the main explanation for a steep sigmoidal transfer function. Thus, sigmoidality in interactions of influence networks (that do not make specific statements about molecular mechanisms) is by default interpreted chemically: namely as manifestation of multimer action with cooperativity, which in the case of gene regulatory networks, would describes the way the TF *X* binds to a promoter [30]. We refer to such explicit mechanistically explained cooperative as ‘*molecular’* cooperativity. This interpretation stems from a conflating of the general dynamical influence network with a chemical reaction network, i.e., from a too literal interpretation of the first derivatives in a formal dynamical system as chemical reaction rates when in reality, the dynamical system equations represent a massive coarse-graining of the chemistry of gene expression which consists of many steps not explicitly considered (chromosome opening, enhanceosome formation, transcription initiation, elongation, RNAs splicing and export, translation, etc). The steps encompass hundreds of elementary chemical reactions.

In fact it is already appreciated that a sigmoidal transfer function in a dynamical system equation, i.e. “*functional* cooperativity”, does not need to reflect underlying *molecular* cooperativity [31]. Several influences, such as the non-ideal physicochemical reaction conditions (molecular crowding, lower than three-dimensional, fractal reaction space, violation of quasi-stationary (Bodenstein) kinetics, stochastic focusing [32] due to small molecule number, etc.) *per se* can all give rise to sigmoidal transfer functions in the absence of molecular cooperativity [31]. Thus, it is important to note that a sigmoidal relationship between rate of a process and abundance of its substrate, manifest e.g., in the form of a Hill function with Hill exponent >1, is not equivalent to the presence of molecular cooperativity.

Separately, the notion of multistability in the absence of cooperativity discussed here shall not be confounded with the phenomenon that in some models stochasticity itself can impart multistability in systems that lack cooperativity and would be monostable (or have a lower number of stable attractors) in the absence of noise [17], [33].

In the above cases of transcriptional regulation involving GATA1, PU.1, Oct4, Nanog, Cdx2, etc. multi-meric reactions have in fact not been reported. To achieve multistability more complex circuits invoking unknown factors have also been proposed [30]. This is reasonable since the canonical bistable or tristable circuits certainly do not exist in isolation.

Here we propose a general approach to integrate the two inputs to each gene that does not depend on the assumption of *molecular* cooperativity or other explicit modeling of a steep sigmoidal transfer function. This is important because nature uses a large variety of interaction modes for reciprocal inhibition of TFs *X* and *Y* involved in binary fate decisions, including protein-protein interaction independent of DNA binding (in the case of GATA1 inhibition of PU.1) [3], [34], [35], [36], [37], [38], [39]; formation of a ternary repressor complex of *X* and *Y* via physical interaction and DNA binding of the complex (Cdx2 inhibition of Oct4 et v.v.) [40]; additional recruitment of complexes that modulate chromatin structure (PU.1 inhibition of GATA1) [34], [41], [42], [43] or repression dominated by binding on each other's promoter (mutual inhibition between Nanog and Cdx2) [28]. Despite the variety of known molecular realization of cross-inhibition, which certainly only represents a partial picture of effective processes, the overall dynamics are similar: the production of a metastable indeterminate state {*X*≈*Y*} that can bifurcate into a bistable regime with the two stable states {*X*>>*Y*} and {*X*<<*Y*}. This convergence to the similar global dynamics lends credence to the notion that a multitude of chemical rate equations map into the same or very similar dynamical systems. Evolution may have realized the same bistable behavior using a variety of molecular implementations. Thus, a general dynamical system description based on influence networks can be of value.

**Figure 1:**
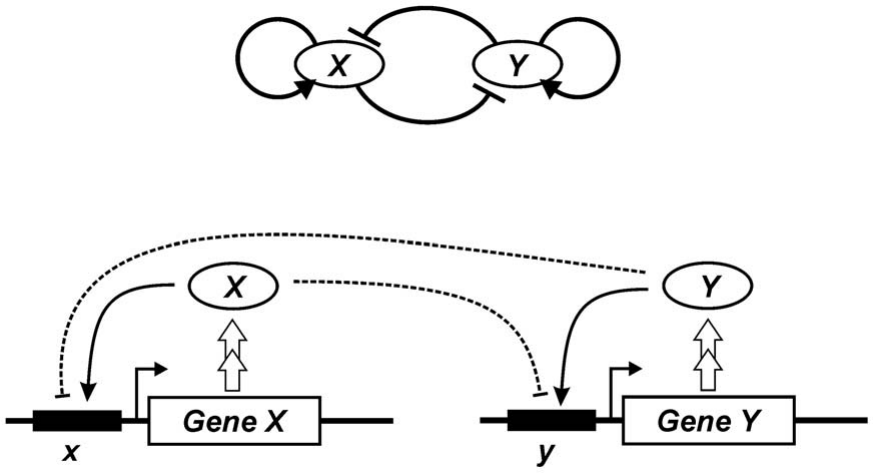
The generic architecture of the self-activation and mutual repression two-gene circuit. Top: coarse-grained circuit scheme for the circuit of two genes X and Y as a *dynamical system*; bottom: molecular mechanism model amenable for a more detailed *chemical reaction kinetics* formalism, indicating the variables for the model due to the distinction between genes/promoters (*x* and *y*) and the transcription factor proteins (*X*, *Y*). Note that the modality for how the two inputs at each promoter, self-activation and cross inhibition is not specified by the scheme.

To formally demonstrate this relationship we formulate here two minimal chemical reaction schemes for the circuit architecture shown in Fig. 1, employing a framework developed originally for the mean-field description of the dynamics and the stochastic simulation of transcription factor expression [33], [44]. Given the lack of knowledge of higher-level interactions we assume essential independence of the two inputs, the auto-stimulation and the cross inhibition. With this approach we arrive at two main conclusions: (a) We show that even without explicit assumption of protein-protein interactions and cooperativity a general dynamical form can be derived in which multi-stability exists. (b) We also find that for some parameter values of the dynamical system that correspond to a symmetry between auto stimulation and inhibition the system can give rise to a degenerate (rather than fixed point) steady-state that corresponds to the indeterminate precursor state.

## Results

### Two reaction kinetics models of the gene regulation circuit

In the two models of the gene circuit shown in Fig. 1 we will focus on the kinetics of the elementary steps that must occur, such as promoter binding of factors *X* and *Y* to their cognate promoter elements, *x* and *y*, respectively. Importantly, we take an unbiased approach, making no assumptions on higher-level relationships, such as the molecular nature of the cross regulation of *X* and *Y*.

#### First model: Independent action of Y and X and autoregulation integrated in effective induction

We first consider that the two transcription factors *X* and *Y* in isolation and model their effective activation (production) kinetics *d*[*X*]/*dt* and *d*[*Y*]/*dt* under the influence of autostimulation without considering mutual repression mechanism. The promoter binding (1.1.), subsequent dissociation (1.2) or self-activation (1.3.), and the degradation (1.4.) reactions for *X* are:
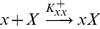
(1.1)

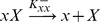
(1.2)

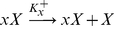
(1.3)

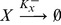
(1.4)(Due to symmetry an analogous set of equations can be written for *Y* and is omitted here).

Here, 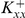, 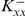 (or, analogously for *Y*, 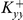, 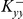) describe the binding and release rates between factor and promoter element, while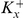, 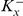
(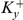, 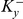) reflect the production and the degradation rates of the transcription factors. The dynamical behavior (rate of change of active levels of the proteins) of the isolated transcription factors is then described by the differential equations, from (1.3. and 1.4), for *X*:
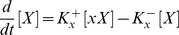
(1.5)

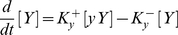
(1.6)where [..] denote concentrations. Assuming that the binding and release processes (in 1.1. and 1.2.) are fastcompared to the production of the proteins (1.3.) and reach chemical equilibrium and taking into account that the total promoter concentrations, [*x* ^0^] and [*y* ^0^], are the sum of respective bound and free promoters, we can eliminate the “complex terms” [*xX*] and [*yY*] and obtain (see Text S1): 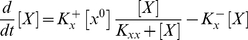
(1.7)

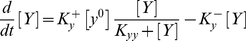
(1.8)where 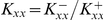 and 
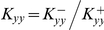
 are the equilibrium constants.

In this first model, the auto-regulation is integrated into the circuit in the following way. We introduce the “effective” activation rates for each transcription factor locus that will absorb the auto-regulation by observing that the above set of chemical reactions, describing the self-activation, can be replaced by only four equivalent reactions:
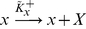
(1.9)

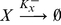
(1.10)

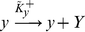
(1.11)

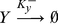
(1.12)where 
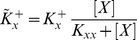
(1.13)

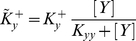
(1.14)are the “effective” activation rates, in the “isolated” self-activation regime, which are nonlinear functions of [*X*], and respectively [*Y*].

Now, let us take the view that the two gene loci interact via the mutual repression mechanism mediated by their encoded proteins that act as trans-repressor, independent of the self-activation that is now encapsulated by the “effective activation” reaction of the loci. The cross-antagonism is only considered through the binding of *X* to *y* and *Y* to *x*, respectively, and no specific mechanism needs to be assumed for the interaction with the components of the self-activation machinery. Then, activation, repression and degradation reactions, for both transcription factors, are then given by:
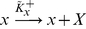
(1.15)

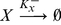
(1.16)

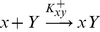
(1.17)

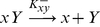
(1.18)(with an analogous set of equations for *y* → *y* + *Y*).

With the “effective” rates of the self-activation processes, 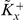 and respectively 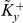, the activation of two proteins follow the these differential equations:
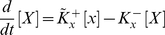
(1.19)

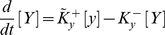
(1.20)Here the free promoters *x* and *y* available for self-activation depend on the concentration of the opposite factors *Y* and *X*, respectively. To express *d*[*X*]/*dt* and *d*[*Y*]/*dt* in the above equations as a function of protein concentrations only, we eliminate [*x*] and [*y*], again, using the assumption that the binding and release mechanism is fast compared to protein production and mass conservation of promoters.

We arrive (see Text S1) at the following differential equations that describe the dynamics of the system when the activation and repression mechanisms are independent:
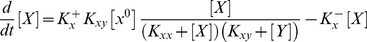
(1.21)

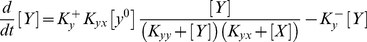
(1.22)where *K_xy_* and *K_yx_* are the equilibrium constants for the ‘cross-binding’ reactions of *X* to *y* and *Y* to *x* (see Text S1). Importantly, this description does not require the explicit introduction of cooperativity or the presence of an extra protein in order to obtain sigmoidality in the system. On the contrary, this model requires only protein monomers that act independently. Yet the cross-term [*X*][*Y*] appears in the denominator and can be interpreted as the formation of a heterodimer 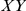 that contributes to cross-inhibition, as is the case of the PU.1 inhibition by GATA1. Clearly, this arises here as a consequence of independent actions of the monomer proteins. We have thus mapped a reaction kinetics formalism into a non-linear dynamical system whose dynamics will be examined later.

#### Second model: Formation of ternary XY-promoter complexes without cooperativity

We now explicitly allow for direct interaction of the proteins and assume the following reaction kinetics for the *X*-locus, in which *Y* binds to *x* and to the complex *xX*:
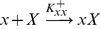
(2.1)

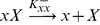
(2.2)

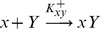
(2.3)

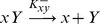
(2.4)

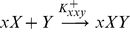
(2.5)

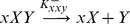
(2.6)

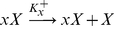
(2.7)

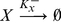
(2.8)(again, with an analogous set of reactions for *Y*).

The Eq. 2.5–2.6 (and the respective mirrored forms for the *Y*-locus) describe the in situ formation of a hetero-dimer *XY* directly on the promoter (ternary complex), without a-priori cooperativity between *X* and *Y*. Since only the reaction of eq. 2.7 (and the respective form for *Y*) contributes to the protein production, the dynamical behavior of the system is described by the following differential equations:
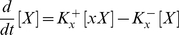
(2.9)

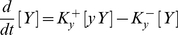
(2.10)Elimination of the promoter-protein complex terms [*xX*] and [y*Y*] (again, assuming equilibrium kinetics for promoter reactionsand using mass conservation for the total promoter concentrations [*x* ^0^] and [*y* ^0^]) we obtain the following differential equations that describe the dynamics of the system (see Text S1):
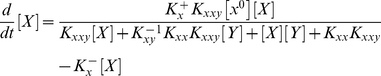
(2.11)

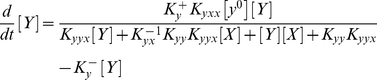
(2.12)where, analogously, *K_xxy_* and *K_yyx_* are equilibrium constants for the cross-binding of *Y* to the *xX* complex and *X* to the *yY* complex, respectively (see Text S1).

Again, the term [*X*][*Y*] indicative of a ‘hetero-dimer’ appears although formation of hetero-dimers was not explicitly assumed. As discussed in the next section, despite distinct chemical interpretation this form is dynamically identical to the result of the first model. Thus, a set of distinct elementary chemical reaction mechanisms of a small network can map to the same non-linear dynamical system.

### Bifurcation dynamics

We now treat the above chemical kinetics descriptions as a generic dynamical system, as discussed in section 1. Thus, for both models (Eqs. 1.21/22 and 2.11/12) we can write the following generic differential equations:
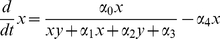
(3.1)

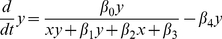
(3.2)where 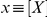, 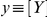 and 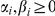, 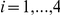. (Note that following customary use, hereafter *x* and *y* are simply the two system variables of a generic dynamical system and do not represent the promoters as above).

To simplify the description we assume a symmetrical system where: 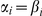, 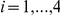. Thus, the simplified system takes the following form:
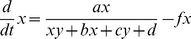
(3.3)

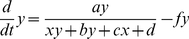
(3.4)The steady states of the above system of differential equations are given by the solutions of the algebraic forms after setting *dx*/*dt*  = 0 and *dy*/*dt*  = 0. For the steady states, one can then easily verify that:
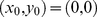
(3.5)

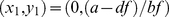
(3.6)

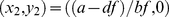
(3.7)

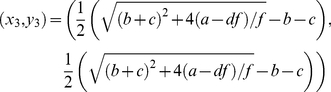
(3.8)are the four steady states of the system with positive values for *x* and *y* (as required for concentrations) if:
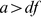
(3.9)To determine the local stability at these steady states we obtain the two eigenvalues *λ* and *μ* for the Jacobian matrix evaluated at these states. For the trivial steady state (*x*_0_, *y*_0_) the eigenvalues are:
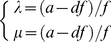
(3.10)This state is always unstable, since 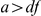 is a necessary condition for the positivity of the solutions.

The second, (*x*_1_, *y*_1_), and the third, (*x*_2_, *y*_2_), steady states have the following eigenvalues:
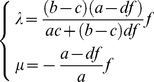
(3.11)Since we always have 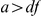, these two states are stable if 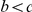 and are unstable for 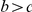.

The eigenvalues of the Jacobian for the forth steady state, (*x*_3_, *y*_3_), are more complicated to calculate analytically. However, one can show numerically that this state is unstable for 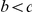, and it becomes stable for 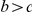.

**Figure 2:**
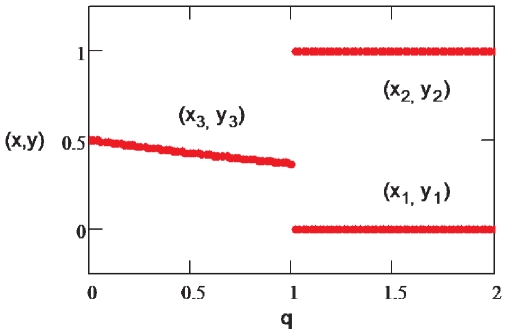
The bifurcation of the system for the bifurcation parameter *q = c/b*. Values are as follows: *a* = *f* = 1,*d* = *b* = 0.5 and 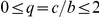. At the critical point *q* = 1 when *c*, which is proportional to cross inhibition, becomes larger than *b*, the system bifurcates from one stable (*x_3_*, *y_3_*) two to stable steady states (*x_1_*, *y_1_*) and (*x_2_*, *y_2_*) (solid lines).

Therefore, the system undergoes a bifurcation by increasing the ratio 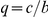. The system changes from a stable equilibrium state (*x*_3_, *y*_3_), when 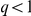, to two stable equilibria (*x*_1_, *y*_1_) and (*x*_2_, *y*_2_), when 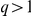. This bifurcation, which is distinct from a pitchfork bifurcation of the toggle-switch [9], [16], [17], is illustrated numerically in Fig. 2. Note the robustness of the stable states (*x*_1_, *y*_1_) and (*x*_2_, *y*_2_) whose positions do not depend on *q*.

**Figure 3:**
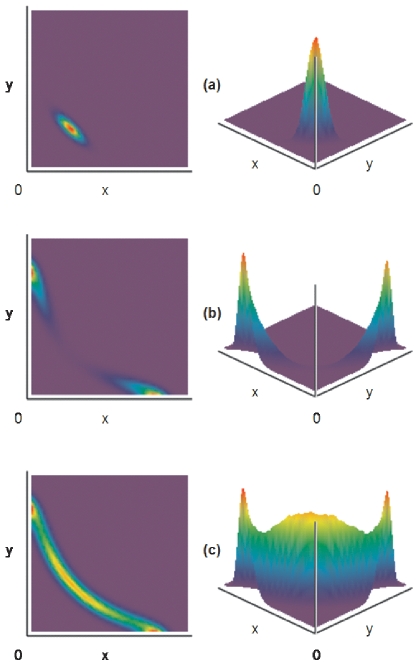
The results of the stochastic simulation of the systemfor three parameter configurations. (a) *b>c*, (b) *c>b* and (c) *c = b*. (see text for details). Colors (or elevation, respectively) represent the steady state probability distribution (cold-to-warm colors for low-to-high probability for finding the circuit at a given position in the *xy*-phase plane).



To gain some insight about the global dynamics of this system [45], [46] in Fig. 3 we present the results of the simulation of the system using the stochastic differential equations, in order to (approximately) visualize non-local dynamics [10], [33], [45], [47], [48]:

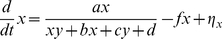
(3.12)

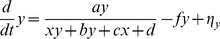
(3.13)where 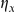 and 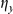 are Gaussian random functions of time, introducing additive noise with a magnitude given by the standard deviation *σ* of the two independent Gaussian processes. (Since the system variables *x* and *y* describe the concentration of protein products, we require that no variable will drop below zero). This probabilistic view affords, to some approximation, the notion of the “relative depths” of attracting steady states in non-integrable systems [45].

In Fig. 3 the density distribution of a trajectory of length 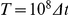, is graphically represented, where 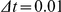, 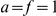, 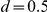 and 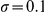, in the space (*x, y*). One can see that for 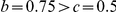, the system has only one (noisy) attractor, corresponding to the stable steady state (*x*_3_, *y*_3_) (Fig. 3a), while for 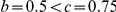, the system exhibits two noisy attractors corresponding to the stable steady states (*x*_1_, *y*_1_), and respectively (*x*_2_, *y*_2_) (Fig. 3b).

An interesting case of the above analysis arises when there is symmetry between *b* and *c* corresponding to the critical bifurcation parameter 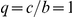 (Fig. 3c). Note from eq. 2.11 and 3.3 that *b* represents self-activation (together with *a*) and *c* is proportional to cross-inhibition. In this case, one finds that the corresponding steady state equations are degenerated, forming a manifold such that:



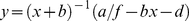
(3.14)Therefore, in this case there is an infinite number of possible steady states (*x*,*y*), all of them satisfying the above equation. Thus, all the (*x, y*) points on this manifold (3.14) satisfy the steady state condition.

**Figure 4:**
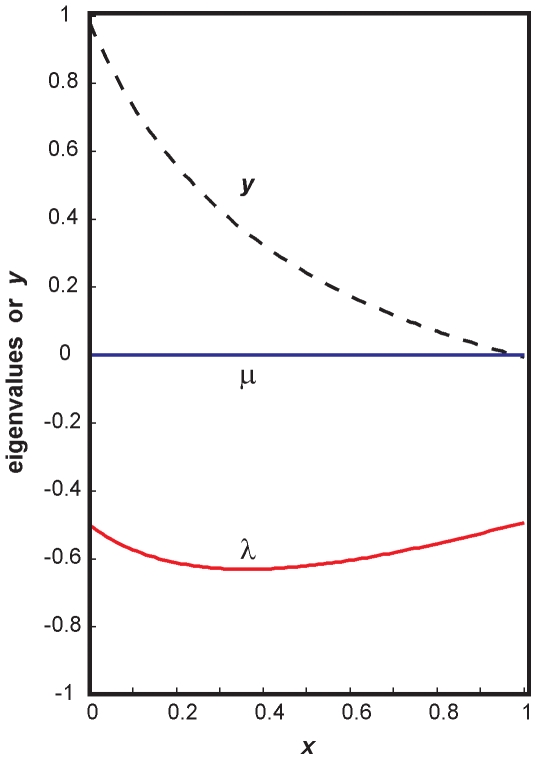
The eigenvalues in the critical region of the bifurcation as a function *x*. Note that *m* is zero for all *x* (blue) and that there is a minimum in *λ* (red) (see text for details). Shown is also the position of the steady state in the *y* dimension as function or *x* (dashed line,  =  stable degenerate manifold, Eq. 3.14).

The eigenvalues of the corresponding Jacobian (obtained by setting  = *b*) depend on the position on this manifold and, using 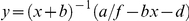, is given by:
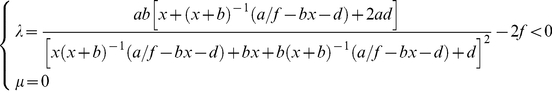
(3.15)for any 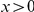, such that 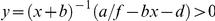. In Fig. 4 the case is shown for 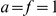, 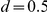 and 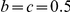. One can see that for all the values 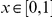 we have 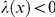 and 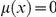. This result shows that in the case of 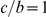 there is a continuous area (a manifold) of critical states, described by the above equation. Biologically, this area may be associated with the set of indeterminate and “primed” state of multipotent progenitor cells in which one can observe intermediate values in the expression of the two transcription factors *X* and *Y* and which form a heterogeneous population with respect to *X* and *Y* levels where individual cells exhibit on average inversely related levels of *X* and *Y* [49], [50], [51], [52]. Fig. 3c. shows the stochastic simulation results (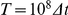, 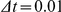, 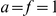, 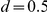, 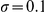) for 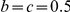, demonstrating that the stability of this region is quite robust to a relatively high noise perturbation 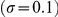. However, a perturbation of the parameters *b* and *c* such that 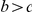 will cause the system to collapse to the stable steady state(*x*_3_, *y*_3_), the progenitor state, whereas a perturbation of the parameters *b* and *c* such that 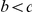 will force the system to choose its lineage since in this regime only two stable attractor states (*x*_1_, *y*_1_) and ( *x*_2_, *y*_2_), exist to either of which the progenitor cell must converge.

## Discussion

Here we analyze two simple models for the gene regulatory circuit that drives binary cell fate decisions, consisting of mutual transcriptional cross-inhibition of two transcription factors *X* and *Y* and self-activation of each. We formulate two distinct models based on elementary chemical kinetics of transcriptional activation controlled by promoter biding events and show that despite fundamental differences in the formalization of the molecular mechanisms they map to the same generic dynamical system that can produce the defining indeterminate state of multipotency and undergo a bifurcation that destabilizes it (form of eqs. 3.3. and 3.4). We report here two novel aspects in the modeling of gene circuits that control resolution of fate indeterminacy during binary cell fate decision.

First, non-linearity and multi-stability arise without assumption of *molecular* cooperativity. While it has been previously noted that sigmoidal rate equations, and hence, bi/multistability, can arise in the absence of such cooperativity if the system is noisy or given particular network structures, or both [17], many theoretical biologists still subliminally equate any sigmoidality in the rate equations, which here we more generally would like to refer to as ‘functional’ cooperativity, with actual ‘molecular’ cooperativity. Without entering into this technical and onomasiological discussion (see introduction) we would like here to rather focus on the mapping of chemical reaction kinetics formalism into a deterministic dynamical system as a source for multistability. It is important to note again the obvious fact that the real molecular mechanisms that govern the dynamics of this gene regulatory circuit are by orders of magnitudes more complex, involving perhaps thousands of steps (including opening of chromatin, formation of initiation complex, transcript elongation, termination and export and the entire system of mRNA maturation and of protein translation) and many more factors, such that detailed molecular models are at the moment not realistic. This is also one reason why the notion of ‘molecular’ cooperativity in mathematical models of mammalian gene regulation is not very meaningful. However, what is certain from observed cell fate decision behavior is the existence of an indeterminate bipotent progenitor state, poised to have equal or similar levels of *X* and *Y*, and the generation of two stable attractor states with reciprocal expression pattern following cell fate decision [6], [7], [8], [9]. This fact is well captured by the general minimal dynamical system of eqs. 3.3. and 3.4. Moreover, the basic architecture of the circuit that involves mutual inhibition and cross-antagonism of the two factors is also widely observed [9], [19] – as far as can be inferred from existing data or derived from perturbation experiments, reporter analysis promoters, protein-DNA binding studies and protein-protein interaction analysis.

Of particular interest and consistent with our conclusion is that despite common general gene circuit architecture and behavior, the molecular implementation can differ considerably. For instance, inhibition of PU.1 by GATA1 occurs via (competitive) protein-protein interaction and does not require GATA1 binding to DNA ([34], [38] and additional refs. in Introduction) whereas inhibition of GATA1 by PU.1 requires PU.1 to bind to DNA and to recruit other proteins that repress the GATA1 promoter, in part via chromatin modification [34], [35]. Conversely, studies on the mutual inhibition between Oct4 and Cdx2 in the early embryo suggest that these two transcription factors that control the first binary cell fate decision, form a repressor complex [40]. In the case of the mutual antagonism between Nanog and Cdx2 the inhibition appears to rely on repressive binding of the cross-antagonist to several distinct sites of the antagonized gene's regulatory region [28]. Thus, while the picture of the actual molecular mechanism is only sketchy, the general statement can be made that evolution has produced a common dynamical behavior scheme for multipotency, as captured by our dynamical system form (eqs. 3.3. and 3.4) and other forms proposed in which mutually inhibitory and self-activating transcription factors are engaged in a circuit [7], [30], [53], [54]. Such circuits typically allow for the existence of a central metastable indeterminacy state and a bifurcation that destabilizes this central symmetric state to produce the asymmetric attractors by employing a variety of molecular realizations. This agrees with the formal notion that distinct chemical kinetics models map into the same dynamical system. The latter may thus be both evolutionarily beneficial and inherently robust.

A similar but distinct dynamical system form that deviates from the form of eqs. 3.3. and 3.4 has been suggested for the GATA1-PU.1 system and shown to predict the observed trajectories of the differentiating cells in the *XY* plane [9]. In that case the central steady state undergoes a pitchfork bifurcation that also forces the cells to adopt either one of the two asymmetric attractor states. Other dynamical systems formulations of this very same GATA.1-PU1 system based on distinct chemical reaction model assumptions produce similar dynamical behaviors and have been compared directly in [55].

The second novelty we report here is the type of bifurcation (at *c* = *b*) that is distinct from the pitchfork bifurcation seen in the toggle switches (with or without self-activation of *X*, *Y*) [9], [16], [17] and is characterized by the existence of a degenerate steady state which forms an attracting manifold *x* = *f*(*y*) (eq. 3.14) in the phase plane due to non-unique solution of the system equations for *dX*/*dt* = *dY*/*dt* = 0. Each point on the manifold is an independent steady state. Because of degeneracy the eigenvalues now can be a function of (*x*, *y*). Specifically, one eigenvalue, *μ*, is zero, and for the other, *λ*, we have *λ* = *f*(*x*,*y*) <0 (for 0≤*x*≤1) whose functional form for the dependence on (*x*, *y*) is state in eq. 3.15. The manifold is attracting, except along itself, that is, there is no “longitudinal” force on this manifold. Therefore every point on it is indifferently stable. However, there is a minimum for *λ* (Fig. 4), *λ_min_* which becomes functionally manifest in the presence of noise, since the point (*x*, *y*) on the manifold for *λ_min_* exerts the highest attracting force. This region correspond to the state of indeterminacy of the progenitor state that can be observed, in which *X* and *Y* are expressed at (on average) similar levels but fluctuate in a inversely correlated manner [49], [50], [51]. This model also would be consistent with the proposal that the indeterminate stem cell state reflects a noise-drive exploratory behavior [56]. In fact, an inverse relationship of abundance of the opposing transcription factors the levels of *X* and *Y* within the same clonal progenitor cell population and despite their noisy fluctuations has recently been observed [50], [52], [57], [58].

The fact that this degenerate manifold exists only if *b* = *c* implies that it is *structurally* unstable that is, it is sensitive to change in control parameters and requires perfect tuning of these parameters. Thus, is the degenerate manifold an artificial mathematical constellation or has it practical relevance?

If the (*x*≈*y*) state on it represents the indeterminate, multipotential stem or progenitor cell, it would in fact capture its natural biological properties: Although such cells are in general considered distinct entities that are observable and isolatable, they are “relatively unstable” in the sense that while identifiable as discrete entity they are short-lived *in vivo* and special differentiation-inhibiting culture conditions are required to maintain the multipotent cells which hence have been referred to as “metastable” [12], [19]. In other words, this formal structural instability may represent the physical instability at a different, namely slower time scale. On the other hand, if we speculate that the unlikely *b* = *c* condition exists given that it would nicely predict the features of the undecided multipotent state, one would have to simultaneously postulate that active regulative fine tuning and maintenance of this *b* = *c* condition may have evolved to ensure the poised state afforded by the degenerate attracting manifold. Such a regulation could be conveyed by the multitudes of inputs from other regulatory factors in which our 2-gene circuit is embedded. At the same time, this structural instability would permit the quick destabilization of the poised stem/progenitor cell state when cells need to undergo a fate decision and commit to a lineage.

One often forgets that the separation of quantities in models into “system variables” and “control parameters”, which is rooted in engineering sciences, is based on the artificial separation of time scales and invokes some higher instance that tunes the control parameters. Such discrete separation of time scales can collapse in complex systems where processes in a continuous range of time scales coexist [59]. Specifically, in a complex, high-dimensional gene molecular network, it is likely that the parameters *b* and *c* are themselves variables (nodes of the network). Then, the critical point *b* = *c* could in principle represent a stable attractor state in the high-dimensional state space, at least in the dimensions of the variable *b* and *c*. This constellation may in fact not be difficult to evolve given that there is selection pressure in metazoan cells to have multipotent metastable states. While there is no experimental evidence for the relative stability for the condition *b*  = *c* yet and the degenerate attractor is of limited mathematical novelty (although to our knowledge not explicitly described) the concept of a degenerate attractor in gene circuits offers a new biological mechanism for producing metastable degenerate states. This can be experimentally verified by single-cell analysis of *X* and *Y* in large populations of bi-potent cells undergoing cell fate decision.

In conclusion, as the detailed molecular characteristics of the chemical mechanisms underlying the interactions in the gene circuit accumulate, the validity of the proposed simple dynamical system can be further evaluated and adjusted as necessary. However, since it is unrealistic to expect a maximally, molecular level fine-grained chemical reaction kinetics model for biological networks, the formulation of generic, simplifying dynamical system equations to which an entire class of chemical reaction networks may converge will remain a central strategy for understanding gene regulatory networks in cell fate control.

## Supporting Information

Text S1 (DOCX)
