# Supplementary material for: A General Model for Binary Cell Fate Decision Gene Circuits with Degeneracy: Indeterminacy and Switch Behavior in the Absence of Cooperativity
Source: PLoS One. 2011 May 19;6(5):e19358. doi: 10.1371/journal.pone.0019358 (PMC3098230; doi:10.1371/journal.pone.0019358)
Supplement: Text S1 — (DOCX) [file pone.0019358.s001.docx]

**SUPPORTING INFORMATION**

**A general model for binary cell fate decision gene circuits with degeneracy: Indeterminacy and switch behavior in the absence of cooperativity**

**Mircea Andrecut^1^, Julianne D. Halley^1#^, David A. Winkler^2,3*^, Sui Huang^1*^**

^1^Institute for Biocomplexity and Informatics, University of Calgary,

Calgary, Alberta, T2N 1N4, Canada

^2^CSIRO Materials Science and Engineering,

Clayton South VIC 3168, Australia

^3^Monash Institute for Pharmaceutical Science, Parkville 3052, Australia

#Present address: Wellcome Trust Centre for Stem Cell Research, Tennis Court Road, Cambridge, CB2 1QR

*email: sui.huang@ucalgary.ca, Dave.Winkler@csiro.au

**DERIVATION OF THE TWO MATHEMATICAL MODELS**

*Here we present in more detail the elementary steps leading to the final differential equations presented in the main text that the reviewers asked us to omit. There is indeed nothing new from a mathematical point of view but for non-mathematicians the following derivation may be of pedagogical value. Unfortunately in today’s interdisciplinary research in life sciences mathematicians usually suppress such obvious derivations - an act which does not help to bridge the increasing gulf of mind-set between the biologists and mathematicians. Specifically, the consideration of fast vs. slow kinetics, and how the former allows for the assumption of chemical equilibrium states that simplify the treatment, is often not explicitly shown. But for the inquisitive biologists the explicit derivation may be useful, notably since it contains some not so standard subtleties in the notation of constants (in Model 1); so we chose to present the elementary derivation here (in a coherent way, step-by-step – including duplicating the steps shown in the main text) .*

**1. The first model: Independent action of Y and X; autoregulation integrated in effective induction** We first consider that the two transcription factors *X*, and *Y* in isolation and model their effective activation (production) kinetics *d*[*X*]/*dt* and *d*[*Y*]/*dt* under the influence of autostimulation without considering mutual repression mechanism. The promoter binding (1.1.), subsequent dissociation (1.2) or self-activation (1.3.), and the degradation (1.4.) reactions for *X* are:

$x+X\underset{\to}{K_{xx}^{+}}xX$ (1.1)

$xX\underset{\to}{K_{xx}^{-}}x+X$ (1.2)

$xX\underset{\to}{K_{x}^{+}}xX+X$ (1.3)

$X\underset{\to}{K_{x}^{-}}\emptyset$ (1.4)

(An analogous set of equations can be written for *Y.*)

Here, $K_{xx}^{+}$, $K_{xx}^{-}$ (or, analogously for *Y*, $K_{yy}^{+}$, $K_{yy}^{-}$) describe the binding and release rates between factor and promoter element, while $K_{x}^{+}$, $K_{x}^{-}$ ($K_{y}^{+}$, $K_{y}^{-}$) reflect the production and the degradation rates of the transcription factors. The dynamical behavior (rate of change of active levels of the proteins) of the isolated transcription factors is then described by the differential equations:

$\frac{d}{dt}[X]=K_{x}^{+}[xX]-K_{x}^{-}[X]$ (1.5)

$\frac{d}{dt}[Y]=K_{y}^{+}[yY]-K_{y}^{-}[Y]$ (1.6)

Assuming that the binding and release mechanism is fast, and reaches equilibrium, compared to the production of the proteins we can write:

$\left[ x \right][X]=K_{xx}[xX]$ (1.7)

$\left[ y \right][Y]=K_{yy}[yY]$ (1.8)

where $K_{xx}=K_{xx}^{-}/K_{xx}^{+}$ and $K_{yy}=K_{yy}^{-}/K_{yy}^{+}$ are the equilibrium constants, and $[X]$ denotes the concentration of species *X* in the system. Also, since the promoters can be in two different states we have:

$\left[ x \right]+[xX]=[x^{0}]$ (1.9)

$\left[ y \right]+[yY]=[y^{0}]$ (1.10)

where $[x^{0}]$ and $[y^{0}]$ are the total concentrations of the promoter. This mass conservation allows us to eliminate the terms for the complexes and solving the above equations we obtain:

$\left[ xX \right]=\left[ x^{0} \right]\frac{[X]}{K_{xx}+[X]}$ (1.11)

$\left[ yY \right]=\left[ y^{0} \right]\frac{[Y]}{K_{yy}+[Y]}$ (1.12)

and therefore:

$\frac{d}{dt}[X]=K_{x}^{+}\left[ x^{0} \right]\frac{[X]}{K_{xx}+[X]}-K_{x}^{-}[X]$ (1.13)

$\frac{d}{dt}[Y]=K_{y}^{+}\left[ y^{0} \right]\frac{[Y]}{K_{yy}+[Y]}-K_{y}^{-}[Y]$ (1.14)

In this first model, this auto-regulation in integrated into the circuit in the following way. We introduce the “effective” activation rates for each transcription factor locus that will absorb the auto-regulation by observing that the above set of chemical reactions, describing the self-activation, can be replaced by only four equivalent reactions:

$x\underset{\to}{\tilde{K}_{x}^{+}}x+X$ (1.15)

$X\underset{\to}{K_{x}^{-}}\emptyset$ (1.16)

$y\underset{\to}{\tilde{K}_{y}^{+}}y+Y$ (1.17)

$Y\underset{\to}{K_{y}^{-}}\emptyset$ (1.18)

where

$\tilde{K}_{x}^{+}=K_{x}^{+}\frac{[X]}{K_{xx}+[X]}$ (1.19)

$\tilde{K}_{y}^{+}=K_{y}^{+}\frac{[Y]}{K_{yy}+[Y]}$ (1.20)

are the ’effective’ activation rates, in the ’isolated' self-activation regime, which are nonlinear functions of $[X]$, and respectively $[Y]$.

Now, let us take the view that the two gene loci interact via the mutual repression mechanism mediated by their encoded proteins that act as trans-repressor, independent of the self-activation which is now encapsulated by the “effective activation’ reaction of the loci. The cross-antagonism is only considered through the binding of *X* to *y* and *Y* to *x*, respectively, and no specific mechanism needs to be assumed for the interaction with the self-activation machinery. Then, activation, repression and degradation reactions, for both transcription factors, are then given by:

$x\underset{\to}{\tilde{K}_{x}^{+}}x+X$ (1.21)

$X\underset{\to}{K_{x}^{-}}\emptyset$ (1.22)

$x+Y\underset{\to}{K_{xy}^{+}}xY$ (1.23)

$xY\underset{\to}{K_{xy}^{-}}x+Y$ (1.24)

with an analogous set of equations for *y* 🡪 *y* + *Y.*

With the ’effective’ rates of the self-activation processes, $\tilde{K}_{x}^{+}$ and respectively $\tilde{K}_{y}^{+}$, the activation of two proteins follow the these differential equations:

$\frac{d}{dt}[X]=\tilde{K}_{x}^{+}[x]-K_{x}^{-}[X]$ (1.25)

$\frac{d}{dt}[Y]=\tilde{K}_{y}^{+}[y]-K_{y}^{-}[Y]$ (1.26)

Here the free promoters *x* and *y* available for self-activation depend on the concentration of the opposite factors *Y* and *X*, respectively. To express *d*[*X*]/*dt* and *d*[*Y*]/*dt* in the above equations as a function of protein concentrations only, we eliminate [*x*] and [*y*], again, using the assumption that the binding and release mechanism is fast compared to protein production and using the mass conservation:

$\left[ x \right][Y]=K_{xy}[xY]$ (1.27)

$\left[ y \right][X]=K_{yy}[yX]$ (1.28)

$\left[ x \right]+[xY]=[x^{0}]$ (1.29)

$\left[ y \right]+[yX]=[y^{0}]$ (1.30)

Eliminating $[x]$ and $[y]$ from these equations we find:

$\left[ x \right]=\left[ x^{0} \right]\frac{K_{xy}}{K_{xy}+[Y]}$ (1.31)

$\left[ y \right]=\left[ y^{0} \right]\frac{K_{yx}}{K_{yx}+[Y]}$ (1.32)

Then, by replacing in eqs. (1.25 and 1.26) $[x]$, $[y]$ with eqs. (1.31 and 1.32) and $\tilde{K}_{x}^{+}$, $\tilde{K}_{y}^{+}$ with eqs. (1.19 and 1.20), we arrive at the following differential equations that describe the dynamics of the system when the activation and repression mechanisms are independent:

$\frac{d}{dt}[X]=K_{x}^{+}K_{xy}\left[ x^{0} \right]\frac{[X]}{\left( K_{xx}+[X] \right)\left( K_{xy}+[Y] \right)}-K_{x}^{-}[X]$ (1.33)

$\frac{d}{dt}[Y]=K_{y}^{+}K_{yx}\left[ y^{0} \right]\frac{[Y]}{\left( K_{yy}+[Y] \right)\left( K_{yx}+[X] \right)}-K_{y}^{-}[Y]$ (1.34)

**2. The Second model: Formation of ternary XY-promoter complexes without cooperativity**

We now allow for direct interaction of the proteins and assume the following reaction kinetics for $X$, in which *Y* binds to *x* and to the complex *xX*:

$x+X\underset{\to}{K_{xx}^{+}}xX$ (2.1)

$xX\underset{\to}{K_{xx}^{-}}x+X$ (2.2)

$x+Y\underset{\to}{K_{xy}^{+}}xY$ (2.3)

$xY\underset{\to}{K_{xy}^{-}}x+Y$ (2.4)

$xX+Y\underset{\to}{K_{xxy}^{+}}xXY$ (2.5)

$xXY\underset{\to}{K_{xxy}^{-}}xX+Y$ (2.6)

$xX\underset{\to}{K_{x}^{+}}xX+X$ (2.7)

$X\underset{\to}{K_{x}^{-}}\emptyset$ (2.8)

(again, with an analogous set of reactions for $Y$.):

The Eq. 2.5-2.6 (and the respective mirrored forms for the *y*-locus) describe the ’in situ’ formation of a ’hetero-dimer’ $XY$ directly on the promoter (ternary complex), without a-priori cooperativity between $X$ and $Y$. Since only the reaction of eq. 2.7 (and the respective form for *Y*) contribute to the protein production, the dynamical behavior of the system is described by the following differential equations:

$\frac{d}{dt}[X]=K_{x}^{+}[xX]-K_{x}^{-}[X]$ (2.9)

$\frac{d}{dt}[Y]=K_{y}^{+}[yY]-K_{y}^{-}[Y]$ (2.10)

Elimination of the promoter-protein complex terms [*xX*]¸and [y*Y*] (again, assuming equilibrium kinetics) yields:

$\left[ x \right][X]=K_{xx}[xX]$ (2.11)

$\left[ x \right][Y]=K_{xy}[xY]$ (2.12)

$\left[ xX \right][Y]=K_{xxy}[xXY]$ (2.13)

and analogously, for *y*, *Y:*

$\left[ yY \right][X]=K_{yyx}[yYX]$. (2.14)

Also, since the promoters can be in four different states we have:

$\left[ x \right]+\left[ xX \right]+[xY]+[xXY]=[x^{0}]$ (2.15)

$\left[ y \right]+\left[ yY \right]+\left[ yX \right]+[yYX]=[y^{0}]$ . (2.16)

Solving the above equilibrium equations for $[xX]$ and $\left[ yY \right]$ we obtain:

$[xX]=\frac{K_{xy}K_{xxy}\left[ x^{0} \right][X]}{K_{xy}K_{xxy}\left[ X \right]+K_{xx}K_{xxy}\left[ Y \right]+K_{xy}\left[ X \right]\left[ Y \right]+K_{xx}K_{xy}K_{xxy}}$ (2.17)

$[yY]=\frac{K_{yx}K_{yxx}\left[ y^{0} \right][Y]}{K_{yx}K_{yyx}\left[ Y \right]+K_{yy}K_{yyx}\left[ X \right]+K_{yx}\left[ Y \right]\left[ X \right]+K_{yy}K_{yx}K_{yyx}}$ (2.18)

Thus, we find the following differential equations which describe the dynamics of the system:

$\frac{d}{dt}[X]=\frac{K_{x}^{+}K_{xxy}\left[ x^{0} \right][X]}{K_{xxy}\left[ X \right]+K_{xy}^{-1}K_{xx}K_{xxy}\left[ Y \right]+\left[ X \right]\left[ Y \right]+K_{xx}K_{xxy}}-K_{x}^{-}[X]$ (2.19)

$\frac{d}{dt}\left[ Y \right]=\frac{K_{y}^{+}K_{yxx}\left[ y^{0} \right]\left[ Y \right]}{K_{yyx}\left[ Y \right]+K_{yx}^{-1}K_{yy}K_{yyx}\left[ X \right]+\left[ Y \right]\left[ X \right]+K_{yy}K_{yyx}}-K_{y}^{-}[Y]$ (2.20)

**3. Bifurcation dynamics**

We now treat the above chemical kinetics descriptions as a generic dynamical system, as discussed in section 1. Thus, for both models we can write the following generic differential equations:

$\frac{d}{dt}x=\frac{\alpha_{0}x}{{xy+\alpha}_{1}x+\alpha_{2}y+\alpha_{3}}-\alpha_{4}x$ (3.1)

$\frac{d}{dt}y=\frac{\beta_{0}y}{{xy+\beta}_{1}y+\beta_{2}x+\beta_{3}}-\beta_{4}y$ (3.2)

where $x\equiv[X]$, $y\equiv[Y]$ and $\alpha_{i},\beta_{i}\geq0$, $i=1,...,4$. (Note that following customary use, hereafter *x* and *y* are simply the two system variables of a generic dynamical system, and do not represent the promoters as in section 2).

To simplify the description we assume a symmetrical system where: $\alpha_{i}=\beta_{i}$, $i=1,...,4$. Thus, the simplified system takes the following form:

$\frac{d}{dt}x=\frac{ax}{xy+bx+cy+d}-fx$ (3.3)

$\frac{d}{dt}y=\frac{ay}{xy+by+cx+d}-fy$ (3.4)

The steady states of the above differential system of equations are given by the solutions of the non-linear algebraic system after setting *dx*/*dt* = 0 and *dy*/*dt* = 0. For the steady states, one can then easily verify that:

$\left( x_{0},y_{0} \right)=\left( 0,0 \right)$ (3.7)

$\left( x_{1},y_{1} \right)=\left( 0,{(a-df)}/{bf} \right)$ (3.8)

$\left( x_{2},y_{2} \right)=\left( {(a-df)}/{bf,0} \right)$ (3.9)

$\left( x_{3},y_{3} \right)=\left( \frac{1}{2}\left( \sqrt{{(b+c)}^{2}+4(a-df)/f}-b-c \right),\frac{1}{2}\left( \sqrt{{(b+c)}^{2}+4(a-df)/f}-b-c \right) \right)$ (3.10)

are the four steady states of the system with positive values (as required for concentrations) for *x* and *y* if:

$a>df$ . (3.11)

To evaluate the local stability of these steady states we compute the eigenvalues, $\lambda$ and $\mu$, of the Jacobian matrix at these steady states:

$J\left( x,y,a,b,c,d,f \right)=\left[ \begin{matrix} \frac{\partial F}{\partial x} & \frac{\partial F}{\partial y} \\ \frac{\partial G}{\partial x} & \frac{\partial G}{\partial y} \end{matrix} \right]=\left[ \begin{matrix} \frac{a(cy+d)}{{(xy+bx+cy+d)}^{2}}-f & \frac{-ax(x+c)}{{(xy+bx+cy+d)}^{2}} \\ \frac{-ay(y+c)}{{(xy+by+cy+d)}^{2}} & \frac{a(cx+d)}{{(xy+by+cx+d)}^{2}}-f \end{matrix} \right]$ (3.12)

A steady state $(x,y)$ is stable if both eigenvalues are negative, $\lambda<0$ and $\mu<0$, and it is unstable if both eigenvalues are positive, $\lambda>0$ and $\mu>0$. The eigenvalues for the trivial steady state $\left( x_{0},y_{0} \right)$ are:

$\left\{ \begin{matrix} \lambda={(a-df)}/f \\ \mu={(a-df)}/f \end{matrix} \right.$ (3.13)

This state is always unstable, since $a>df$ is a necessary condition for the positivity of the solutions.

The second, $\left( x_{1},y_{1} \right)$, and the third, $\left( x_{2},y_{2} \right)$, steady states have the following eigenvalues:

$\left\{ \begin{matrix} \lambda=\frac{\left( b-c \right)(a-df)}{ac+(b-c)df}f \\ \mu=-\frac{a-df}{a}f \end{matrix} \right.$ (3.14)

Since we always have $a>df$, these two states are stable if $b<c$ and are unstable for *b* > *c*.

The eigenvalues of the Jacobian for the forth steady state, $\left( x_{3},y_{3} \right)$, are more complicated to calculate analytically. However, one can show numerically that this state is unstable for $b<c$, and it becomes stable for $b>c$.

Therefore, the system undergoes a bifurcation by increasing the ratio $q=c/b$. The system changes from a stable equilibrium state ${(x}_{3},y_{3})$, when $q<1$, to two stable equilibria ${(x}_{1},y_{1})$ and ${(x}_{2},y_{2})$, when $q>1$. This bifurcation, which is distinct from a pitchfork bifurcation of the toggle switch [1,2,3], is illustrated numerically in Fig. 2 in the main text. Note the robustness of the stable states ${(x}_{1},y_{1})$ and ${(x}_{2},y_{2})$ whose positions do not depend on $q$.

To gain insight about the global dynamics of t his system [4,5] in Fig. 3 we present the results of the simulation of the system using the stochastic differential equations, in order to visualize non-local dynamics [4,6,7,8,9]:

$\frac{d}{dt}x=\frac{ax}{xy+bx+cy+d}-fx+\eta_{x}$ (3.15)

$\frac{d}{dt}y=\frac{ay}{xy+by+cx+d}-fy+\eta_{y}$ (3.16)

where $\eta_{x}$ and $\eta_{y}$ are Gaussian random functions of time. introducing additive noise with a magnitude given by the standard deviation $\sigma$ of the two independent Gaussian processes. (Since the system variables $x$ and $y$ describe the concentration of protein products, we require that no variable will drop below zero). This probabilistic view affords the notion of the “relative depths” of attracting steady states [4].

In Fig. 3 the density distribution of a trajectory of length $T={10}^{8}\Delta t$, is graphically represented, where $\Delta t=0.01$, $a=f=1$, $d=0.5$ and $\sigma=0.1$, in the space $(x,y)$. One can see that for $b=0.75>c=0.5$, the system has only one (noisy) attractor, corresponding to the stable steady state ${(x}_{3},y_{3})$ (Fig. 3aa), while for $b=0.5<c=0.75$, the system exhibits two noisy attractors corresponding to the stable steady states ${(x}_{1},y_{1})$, and respectively ${(x}_{2},y_{2})$ (Fig. 3b).

An interesting case of the above analysis arises when there is symmetry between *b* and *c* corresponding to the critical bifurcation parameter $q=c/b=1$ (Fig. 3c). Note from eq. 2.18 and 3.3 that *b* represents self-activation (together with *a*) and *c* is proportional to cross-inhibition. In this case, one finds that the corresponding steady state equations are degenerated, such that:

$xy+b\left( x+y \right)+d=a/f$ (3.17)

Therefore, in this case there is an infinite number of possible steady states (*x*,*y*), all of them satisfying the equation:

$y=\left( x+b \right)^{-1}(a/f-bx-d)$ (3.18)

Thus, all the $(x,y)$ points on this manifold satisfy the steady state condition.

The eigenvalues of the corresponding Jacobian (obtained by setting $c=b$ ) depend on the position on this manifold and, using $y=\left( x+b \right)^{-1}(a/f-bx-d)$, is given by:

$\left\{ \begin{matrix} \lambda=\frac{ab\left[ {x+\left( x+b \right)}^{-1}\left( a/f-bx-d \right) \right]+2ad}{\left[ x\left( x+b \right)^{-1}\left( a/f-bx-d \right)+bx+b\left( x+b \right)^{-1}\left( a/f-bx-d \right)+d \right]^{2}}-2f<0 \\ \mu=0 \end{matrix} \right.$ (3.19)

for any $x>0$, such that $y=\left( x+b \right)^{-1}\left( a/f-bx-d \right)>0$.

(See main text for the rest.)

**REFERENCES**

1. Gardner TS, Cantor CR, Collins JJ (2000) Construction of a genetic toggle switch in Escherichia coli. Nature 403: 339-342.

2. Lipshtat A, Loinger A, Balaban NQ, Biham O (2006) Genetic toggle switch without cooperative binding. Physical Review Letters 96: -.

3. Huang S, Guo YP, May G, Enver T (2007) Bifurcation dynamics of cell fate decision in bipotent progenitor cells. Dev Biol 305: 695-713.

4. Nicolis G (1986) Dissipative systems. Rep Prog Phys 49 873-949

5. Wang J, Xu L, Wang EK, Huang S (in press) The “potential” landscape of genetic circuits imposes the arrow of time in stem cell differentiation Biophys J.

6. Sneppen K, Giovanni Z, editors (2005) Physics in Molecular Biology. 1 ed. Cambridge: Cambridge University Press.

7. Andrecut M, Cloud D, Kauffman SA (2008) Monte Carlo simulation of a simple gene network yields new evolutionary insights. J Theor Biol 250: 468-474.

8. Macarthur BD, Ma'ayan A, Lemischka IR (2009) Systems biology of stem cell fate and cellular reprogramming. Nat Rev Mol Cell Biol 10: 672-681.

9. Wang J, Xu L, Wang E, Huang S (2010) The potential landscape of genetic circuits imposes the arrow of time in stem cell differentiation. Biophys J 99: 29-39.
